# Type 2 diabetes seems not to be a risk factor for the carpal tunnel syndrome: a case control study

**DOI:** 10.1186/1471-2474-15-346

**Published:** 2014-10-14

**Authors:** Steven H Hendriks, Peter R van Dijk, Klaas H Groenier, Peter Houpt, Henk JG Bilo, Nanne Kleefstra

**Affiliations:** Diabetes Centre, Isala, Zwolle The Netherlands; Department of General Practice, University of Groningen, Groningen, The Netherlands; Department of Plastic Surgery, Isala, Zwolle The Netherlands; Department of Internal Medicine, University of Groningen, Groningen, The Netherlands; Langerhans, Medical Research Group, Zwolle, The Netherlands

**Keywords:** Carpal tunnel syndrome, Type 2 diabetes mellitus, Risk factor

## Abstract

**Background:**

Previous studies have shown that the carpal tunnel syndrome seems to occur more frequently in patients with diabetes mellitus and might be associated with the duration of diabetes mellitus, microvascular complications and degree of glycaemic control. Primary aim was to determine if type 2 diabetes can be identified as a risk factor for carpal tunnel syndrome after adjusting for possible confounders. Furthermore, the influence of duration of diabetes mellitus, microvascular complications and glycaemic control on the development of carpal tunnel syndrome was investigated.

**Methods:**

Retrospective, case–control study using data from electronic patient charts from the Isala (Zwolle, the Netherlands). All patients diagnosed with carpal tunnel syndrome in the period from January 2011 to July 2012 were included and compared with a control group of herniated nucleus pulposus patients.

**Results:**

A total of 997 patients with carpal tunnel syndrome and 594 controls were included. Prevalence of type 2 diabetes was 11.5% in the carpal tunnel syndrome group versus 7.2% in the control group (Odds Ratio 1.67 (95% confidence interval 1.16-2.41)). In multivariate analyses adjusting for gender, age and body mass index, type 2 diabetes was not associated with carpal tunnel syndrome (OR 0.99 (95% CI 0.66-1.47)). No differences in duration of diabetes mellitus, microvascular complications or glycaemic control between groups were detected.

**Conclusion:**

Although type 2 diabetes was more frequently diagnosed among patients with carpal tunnel syndrome, it could not be identified as an independent risk factor.

**Electronic supplementary material:**

The online version of this article (doi:10.1186/1471-2474-15-346) contains supplementary material, which is available to authorized users.

## Background

Carpal tunnel syndrome (CTS) is one of the most frequent compression neuropathies of the upper limb[1]. Due to entrapment of the median nerve between the flexor tendons of the hand in the carpal tunnel symptoms, like tingling and noctural burning pain, occur[1]. The combination of these clinical symptoms together with positive signs by physical examination and nerve conduction studies (NCS) is the most valid way of diagnosing CTS[1].

The prevalence of CTS in the general population is approximately 2.1% for men and 3.0% for women[2]. Obesity, hypothyroidism, pregnancy, rheumatoid arthritis, osteoarthritis and occupational factors like repetitive work are identified as the main risk factors for CTS[1]. In addition, diabetes mellitus (DM) is also considered as a risk factor[3–7]. Furthermore some researchers found a higher incidence of CTS in patients with pre-diabetes[8], nevertheless screening for DM in patients with CTS is not recommended[9]. Literature suggests also a relationship between HbA1c, duration of DM, microvascular complications and CTS[10–12]. However, many studies investigated DM as a risk factor for musculoskeletal disorders of the hand and shoulder in general and not for CTS in particular[11, 13].

Aim of the present study was to determine if type 2 diabetes mellitus (T2DM) can be identified as a risk factor for CTS. Furthermore, we investigated the influence of diabetes duration, glycaemic control and presence of microvascular complications on the development of CTS.

## Methods

### Study population and design

This study was conducted at the Isala, a general hospital with a catchment area of 800.000 inhabitants in the North East of the Netherlands. All patients with severe symptoms of CTS or with symptoms which could not be treated by a general practitioner with a conservative approach (i.e. watchful waiting, a brace or corticosteroid injections) and who were referred to the outpatient clinic of the Isala between January 2011 and July 2012 were identified. The diagnosis CTS was defined as the presence of classical symptoms for CTS consisting of nocturnal pain associated with tingling and numbness in the distribution of median nerve in the hand. Patients were only included when NCS were performed during the diagnostic process of CTS, irrespective of the outcome of the NCS. NCS were executed by a neurologist and consisted of 1 motor and 2 sensory conduction tests. A comparison was made of the distal motor latencies from the median nerve to the second lumbrical and from the ulnar nerve to the second interosseous muscle with equal distances. Furthermore, a comparison was made of the sensory conduction of the median nerve with the ulnar nerve between wrist and digit 4 and of the sensory conduction of the median with the radial nerve between wrist and thumb. If two of three tests were abnormal, the diagnosis of CTS was electrophysiologically confirmed. All nerve conduction studies were performed with a Synergy system of Viasys Healthcare from 2005 without controlling for the hand temperature. Patients with an (ipsi and/or contralateral) recidive of a previous CTS were excluded.

In order to compare the prevalence of T2DM among patients with CTS with the general population, a control group was formed consisting of operatively treated herniated nucleus pulposus (HNP) patients. HNP patients were chosen assuming that HNP patients would be derived from about the same age category as the CTS group and because it is a frequently diagnosed disease which makes it possible to build a control group consisting of enough patients. Finally only operated patients were chosen because DM status and BMI data are known for the majority of operated patients in our hospital. Patients in the control group were treated in the same period as the CTS group. They were excluded when they received an operation because of a relapse or had received the diagnosis CTS in the past.

### Data sources

Patients, both CTS cases and the HNP controls, were identified using diagnosis-treatment-combination (DTC) codes, which are used in the Netherlands for both hospital registration and health insurance declaration purposes. Each DTC code contains information about the specialty of the treating physician, the patient’s diagnosis and the type of treatment provided. CTS patients were identified using codes 0304.350, 0304.351 and 0330.0801 and HNP patients using the codes 0308.2530, 0308.2550, 0308.2555. The DTC codes for DM and diabetic retino-pathie were used to identify patients with T2DM who are treated in our clinic.

Demographic and clinical data of the CTS and HNP patients treated in the Isala was derived from the individual electronic patient database of the Isala. Furthermore this database was used to verify the CTS and HNP diagnoses. The diabetes specific database of our Diabetes Centre was used for identifying and further detailing of information regarding patients with T2DM who are treated in primary care. General practitioners in our region, receive bench mark information from our Diabetes Centre and therefore we gather data of all primary care treated patients with T2DM on a yearly basis. This database is used in our Diabetes Centre for study purposes. Permission to use the CTS and HNP related data was given by de data management centre of the Isala.

### Variables

Age, gender, body mass index (BMI) and blood pressure were documented for all patients. Date of CTS diagnosis, CTS side, result of nerve conduction studies and type of treatment were recorded in the database for CTS patients. Duration of DM, types of medication, HbA1c, renal function and the occurrence of albuminuria, retinopathy and neuropathy were documented for all T2DM patients.

### Outcome

Primary outcome was the prevalence of T2DM in both groups. Secondary outcomes were duration of DM, microvascular complications (albuminuria, retinopathy and neuro-pathie together) and glycaemic control in relation to CTS development.

### Statistical analysis

Statistical analysis was carried out in SPSS (version 20) using logistic regression, adjusting for confounding variables. Multiple imputation (10 imputed datasets) was used for missing BMI values. The sample size required for the control group to detect a difference in T2DM prevalence of 5%, assuming a CTS group size of 900 patients, a power of 0.8, and an alpha of 0.05 was 459.

### Ethical approval

This study is performed in accordance with the Declaration of Helsinki. According to Dutch guidelines this research does not fall under the scope of the Medical Research Involving Human Subjects Act, and therefore this study does not need a formal approval of an accredited METC (The Medical Ethics Committee of the Isala).

## Results

**Figure 1:**
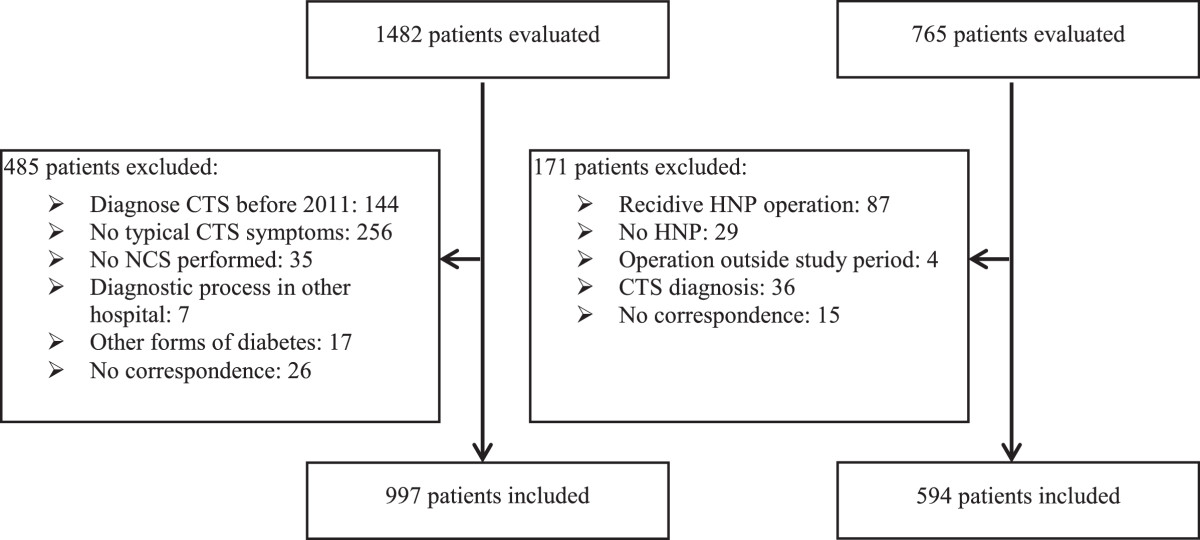
**Flowchart inclusion.**

A total of 1482 patients with a DTC for CTS and 765 patients with a DTC for HNP were identified of which 485 CTS patients and 171 HNP controls did not meet the inclusion criteria (Figure 1). Eventually, 997 persons with CTS and 594 controls were included.

### Primary outcome

The results of the univariate analyses are presented in Table 1. The prevalence of T2DM was 11.5% in the CTS group and 7.2% in the control group (Odds Ratio (OR) 1.67 (95% confidence interval (CI) 1.16-2.41)). The percentage of female patients was significantly higher in the CTS group compared with the control group (OR 2.54 (95% BI 2.06-3.14), p <0.001). The mean age was 55.7 (±15.2) years in the CTS group and 49.3 (±13.0) years in the control group (p <0.001). Furthermore, the mean BMI was also higher in the CTS group (p <0.001).

**Table 1:** **Results of univariate analyses between the CTS group and the control group**

Variables	CTS group	Control group	p-value	OR (95% CI)
	(n =997)	(n =594)		
T2DM	115 (11.5)	43 (7.2)	0.006	1.67 (1.16-2.41)
Gender (female)	710 (71.2)	293 (49.3)	<0.001	2.54 (2.06-3.14)
Age (years)	55.7 ± 15.2	49.3 ± 13.0	<0.001	1.03 (1.02-1.04)
BMI (kg/m^2^)^a^	28.3 ± 5.4	26.7 ± 4.6	<0.001	1.06 (1.04-1.09)
Systolic RR (mmHg)	138.8 ± 21.4	138.6 ± 20.6	0.870	1.00 (0.99-1.01)
Age DM patients (years)	65.6 ± 12.7	60.1 ± 13.7	0.021	1.03 (1.01-1.06)
Gender DM patients (female)	74 (64.3%)	21 (48.8%)	0.078	1.89 (0.93-3.84)
BMI DM patients (kg/m2)	31.2 ± 5.7	29.1 ± 5.1	<0.001	1.07 (1.04-1.10)
Median diabetes duration (months)^b^	103 (55–172)	80 (47–166)	0.389	1.00 (1.00-1.01)
HbA1c (% (mmol/mol))^c^	7.1 ± 3.2 (54 ± 11)	7.3 ± 3.2 (56 ± 12)	0.567	0.99 (0.95-1.03)
Microvascular complications^d^	46 (40.0%)	13 (30.2%)	0.402	0.68 (0.28-1.67)
Metformin	69 (60.0%)	26 (60.5%)	0.582	1.15 (0.70-1.89)
SU-derivate	34 (29.6%)	7 (16.3%)	0.09	2.19 (0.89-5.40)
Insulin	52 (45.2%)	18 (41.9%)	0.673	1.17 (0.57-2.37)

Values are depicted as number (%), mean ± SD or median with 25^th^ and 75^th^ percentile.

^a^Imputation was used for 229 patients with a missing value for BMI in the CTS group and for 12 controls.

^b^2 missing values in the CTS group and 16 in the control group.

^c^6 missing values in the CTS group and 20 in the control group.

^d^12 missing values in the CTS group and 19 the control group.

Five hundred sixty-seven (56.9%) patients had a bilateral CTS and 430 patients had an unilateral CTS of whom 252 (58.6%) right-sided. In 921 (92.4%) subjects diagnosis was confirmed by NCS. Treatment consisted in 18.3% of conservative treatment, in 78.1% of operation and was not well documented in 3.6% of the patients. Four hundred twenty-one patients in the control group had a herniated disc at the lumbar spine level and 173 patients had a herniated disc at the cervical level.

Table 2 displays the results of the multivariate analyses. In model 1 (adjusting for gender) and in model 2 (adjusting for both gender and BMI) T2DM was a significant predictor for CTS. However, when age was added (model 3), T2DM disappeared as a significant predictor (OR 0.99 (95% BI 0.66-1.47)). Gender, BMI and age remained significant predictors for CTS in this final model. The same results were obtained when only patients with CTS confirmed by nerve conduction studies were included (data not shown).

**Table 2:** **Multivariate analysis of T2DM as an risk factor for CTS adjusting for gender, age and BMI**

model (n =1591)	B	p-value	OR (95% CI)
*Model 1*			
T2DM	0.571	0.003	1.77 (1.22-2.58)
Gender^a^	0.945	<0.001	2.57 (2.08-3.18)
*Model 2*			
T2DM	0.389	0.048	1.48 (1.00-2.17)
Gender^a^	0.953	<0.001	2.59 (2.09-3.22)
BMI^b^	0.060	<0.001	1.06 (1.04-1.09)
*Model 3*			
T2DM	-0.014	0.946	0.99 (0.66-1.47)
Gender^a^	0.976	<0.001	2.65 (2.13-3.31)
Age	0.034	<0.001	1.03 (1.03-1.04)
BMI^b^	0.064	<0.001	1.07 (1.04-1.09)

B = coefficient of logistic regression.

^a^Male gender is reference category.

^b^Imputation was used for 229 patients with a missing value for BMI in the CTS group and for 12 controls.

### Secondary outcomes

Analyses of the subgroups with T2DM are also displayed in Table 1. The CTS group contained 115 patients with T2DM versus 43 patients with T2DM in the control group. Patients in the CTS group were significantly older and had a higher BMI. Duration of DM, presence of microvascular complications and glycaemic control did not differ between the two groups.

## Discussion

In this study we investigated the relationship between T2DM and CTS. Although T2DM was more frequently diagnosed among patients with CTS, it could not be identified as a risk factor after adjusting for BMI, gender and age. However, BMI, gender and age remained having a relationship with CTS. No associations were found between the duration of DM or microvascular complications and CTS. In addition, as demonstrated previously, we were unable to find a relationship between glycaemic control and CTS development[11].

This is the first study to investigate the relation between CTS and T2DM in a secondary care setting. Strengths of this study include the use of different data sources to overcome limitations of the retrospective design (i.e. misclassification of CTS diagnosis and missing data). In addition, the use of different data sources enabled us to make a certain diagnosis of CTS and T2DM.

Several limitations should be mentioned. First, cases and controls were only enrolled in a secondary care setting and thereby mostly severe cases were included. Thus, less severe CTS patients treated with conservative treatment by general practitioners did not participate in the current study. Therefore the generalizability of our results is limited to secondary care. In addition, external validity is also limited due to the fact that the studied population consisted of only ~1% of the DM and ~11% of the CTS patients of the total amount of DM and CTS patients in our region. Moreover, although both the operated CTS and HNP patients had the same kind of pre-operative screening in which the presence of DM was assessed, we cannot exclude the possibility of more intensive screening in one of the two groups which may have resulted in a different DM prevalence within and between the two groups. Furthermore, differential misclassification bias could have occurred because conservatively treated CTS patient have had no preoperatively consultation. The choice to use surgery-treated HNP patients as a control population is also a topic for debate. Smaller studies have described a relationship between DM and HNP, so although inconclusive, the prevalence of T2DM could be higher among these persons than in the general population[14]. Additionally, as overweight is identified as a risk factor for the occurrence of lumbar disc herniation, and overweight is also is a major risk factor for T2DM, this could have resulted in a higher prevalence of DM in the HNP population compared with the general population[15, 16]. Therefore, shared risk factors, such as BMI, should be taken into account when interpreting the results of our study. At last, it should be noticed that, due to the amount of missing data any relevant relationships cannot be excluded based on the present study.

Similar to our results, a study among 156 CTS patients and 473 age and sex matched controls derived from a Dutch population register could not find a relation between DM and CTS[17]. Furthermore, a study from Greece found an identical prevalence of DM in a CTS population as compared to control subjects from the general population[18]. On the other hand a significant relation was found in a larger cohort of 3391 CTS patients and 13.564 matched controls (OR 1.51 (95% BI 1.24-1.84))[4]. A systematic review indicated DM as a risk factor for CTS (OR 2.2 (95% BI 1.5-3.1))[3]. However, not all studies included in this review controlled for age differences in multivariate models, which was found to be an important confounding variable in the present study.

Taken together, it could be hypothesized that BMI and age, both well known risk factors for T2DM, are important risk factors for CTS as well, and may explain the relationship found between T2DM and CTS in other studies. Despite the limitations of the present study, i.e. choice of control group, limited generalizability due to the secondary setting, magnitude of missing data and small sample size, this is a hypothesis worth further testing.

## Conclusion

Although type 2 diabetes was more frequently diagnosed among patients with carpal tunnel syndrome, it could not be identified as an independent risk factor. BMI and age, which are well known risk factors for T2DM, are important risk factors for CTS as well and may confound the previously found relationship between type 2 diabetes and CTS.

## Authors’ original submitted files for images

Below are the links to the authors’ original submitted files for images. Authors’ original file for figure 1

## Abbreviations

CTS: Carpal tunnel syndrome
DM: Diabetes mellitus
T2DM: Type 2 diabetes mellitus
NCS: Nerve conduction studies
HNP: Herniated nucleus pulposus
DTC: Diagnosis-treatment-combination
BMI: Body mass index
OR: Odds ratio
CI: Confidence interval.
