# Supplementary material for: Type 2 diabetes seems not to be a risk factor for the carpal tunnel syndrome: a case control study
Source: BMC Musculoskelet Disord. 2014 Oct 14;15:346. doi: 10.1186/1471-2474-15-346 (PMC4210523; doi:10.1186/1471-2474-15-346)
Supplement: Supplementary file 1 — Authors’ original file for figure 1 [file 12891_2014_2284_MOESM1_ESM.pdf]

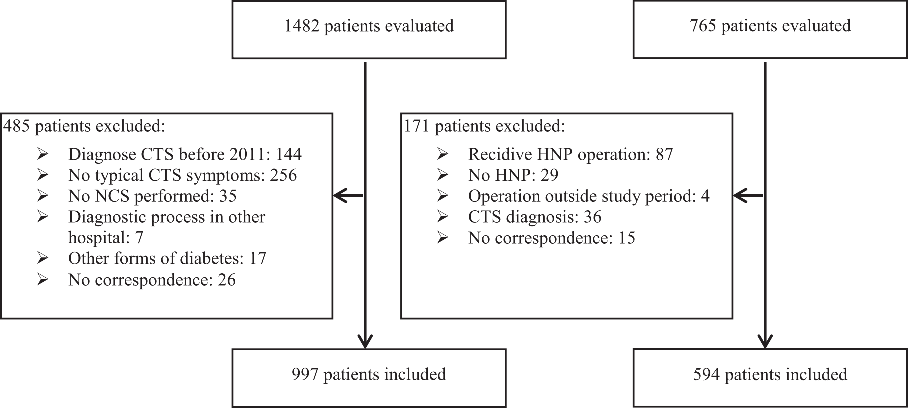
1482 patients evaluated
765 patients evaluated
485 patients excluded:
Diagnose CTS before 2011: 144
No typical CTS symptoms: 256
No NCS performed: 35
Diagnostic process in other hospital: 7
Other forms of diabetes: 17
No correspondence: 26
171 patients excluded:
Recidive HNP operation: 87
No HNP: 29
Operation outside study period: 4
CTS diagnosis: 36
No correspondence: 15
997 patients included
594 patients included
